# Bacterial community analysis in upflow multilayer anaerobic reactor treating high‐solids organic wastes

**DOI:** 10.1002/btpr.2540

**Published:** 2017-09-06

**Authors:** Si‐Kyung Cho, Kyung‐Won Jung, Dong‐Hoon Kim, Joong‐Chun Kwon, Umer Zeeshan Ijaz, Seung Gu Shin

**Affiliations:** ^1^ Dept. of Biological and Environmental Science Dongguk University, 32 Dongguk‐ro, Ilsandong‐gu Goyang Gyeonggi‐do Republic of Korea; ^2^ Department of Earth and Environmental Sciences, Korea University, 145 Anam‐ro Seongbuk‐gu, Seoul 02841 Republic of Korea; ^3^ Dept. of Civil Engineering Inha University, 100 Inharo Nam‐gu Incheon Republic of Korea; ^4^ Ecodigm, 10‐6, 339 Expo‐ro Yuseong‐gu Daejeon Republic of Korea; ^5^ Infrastructure and Environment Division School of Engineering, University of Glasgow Glasgow G12 8LT UK; ^6^ Department of Energy Engineering Gyeongnam National University of Science and Technology (GNTECH) Jinju Republic of Korea

**Keywords:** anaerobic digestion, food waste leachate, plug flow, 16S rRNA gene, 454 pyrosequencing

## Abstract

A novel anaerobic digestion configuration, the upflow multi‐layer anaerobic reactor (UMAR), was developed to treat high‐solids organic wastes. The UMAR was hypothesized to form multi‐layer along depth due to the upflow plug flow; use of a recirculation system and a rotating distributor and baffles aimed to assist treating high‐solids influent. The chemical oxygen demand (COD) removal efficiency and methane (CH_4_) production rate were 89% and 2.10 L CH_4_/L/d, respectively, at the peak influent COD concentration (110.4 g/L) and organic loading rate (7.5 g COD/L/d). The 454 pyrosequencing results clearly indicated heterogeneous distribution of bacterial communities at different vertical locations (upper, middle, and bottom) of the UMAR. Firmicutes was the dominant (>70%) phylum at the middle and bottom parts, while Deltaproteobacteria and Chloroflexi were only found in the upper part. Potential functions of the bacteria were discussed to speculate on their roles in the anaerobic performance of the UMAR system. © 2017 The Authors Biotechnology Progress published by Wiley Periodicals, Inc. on behalf of American Institute of Chemical Engineers *Biotechnol. Prog*., 33:1226–1234, 2017

## Introduction

Anaerobic digestion (AD) involves a series of sophisticated microbial reactions including harmonious competition and syntrophy for their substrates during the biotransformation processes of hydrolysis, acidogenesis, and methanogenesis. AD has been considered as the most environmentally friendly disposal method for various organic wastes due to the following advantages: (i) reduction of waste volume, (ii) production of a nutrient‐rich final product, and (iii) generation of energy‐rich biogas in the form of methane (CH_4_).^1^ The versatile convertibility of biogas to other useful energy forms such as heat, electricity, and vehicle fuel makes AD one of the most important renewable energy sources. The importance of AD undoubtedly seems to be growing around the world because most countries aim to achieve energy policy goals of significantly increasing the share of renewable energy production.^2^

AD performance is directly related to the concentration and activity of microorganisms. Consequently, various anaerobic bioreactors have been developed and optimized towards higher retention of viable microorganisms and better contact with substrate, resulting in ‘high‐rate’ systems.^3^ The anaerobic contact reactor, anaerobic filter reactor, fluidized bed reactor, expanded bed reactor, anaerobic membrane bioreactor, and upflow anaerobic sludge blanket reactor (UASBr) are examples of the high‐rate systems. Among these, UASBr, which uses an up‐stream flow scheme, has been considered one the most successful systems and has achieved worldwide popularity. The success of UASBr and its modified configurations, such as expanded granular sludge bed and internal circulation reactor, is attributable to the formation of granules to support the dense sludge bed with high microbial diversity inside the reactor.^4^ However, the necessity of a long start‐up period and restriction to applying high‐solids wastes have been considered as their limitations.^5^ Thus, an alternative reactor configuration that does not require granule formation could circumvent these problems by allowing migration of solids while operated at high organic loading rates (OLRs).^6^

Characterization of the microbial community structure is critical for the fundamental understanding of the digestion efficiency.^7^ To date, various molecular biological tools based on 16S rRNA genes, such as the polymerase chain reaction (PCR), denaturing gradient gel electrophoresis, fluorescence *in situ* hybridization, and restriction fragment length polymorphism have been applied to assess AD reactors.^7^, ^8^, ^9^, ^10^ State‐of‐the‐art next‐generation sequencing (NGS) such as 454 pyrosequencing provides high‐throughput sequencing for deeper taxonomic resolution of microbial communities at time‐ and cost‐effective scales.^11^ However, although there are increasing number of reports in the literature on microbial community analysis in AD processes, still relatively limited information is available on the microbial community structures in high‐rate AD systems using NGS.^4^, ^12^, ^13^, ^14^

There is growing evidence of spatial distribution of the anaerobic consortia within high‐rate AD processes.^4^, ^6^ Although Xing *et al*.^6^ claimed that the spatial stratification of anaerobic microbes has contributed to the diversity of the anaerobic consortia inside the reactor and consequently to the performance of the anaerobic process, it is still highly underexplored whether this tendency is replicated in other types of high‐rate AD reactors.^15^ The level of stratification would be dependent on various factors such as the reactor configuration.

In this study, a novel high‐rate anaerobic system, named as an upflow multi‐layer anaerobic reactor (UMAR), is suggested and tested to treat high‐solids organic wastes. The key feature of UMAR is its vertically multi‐layered microbial structure via upward plug flow, which is hypothesized to allow migration of solids and to ensure high AD performance by allowing enhanced microbial functions at different vertical locations of the reactor (upper, middle, bottom). To test this hypothesis, a lab‐scale (60 L) UMAR was continuously operated for over 200 days to evaluate CH_4_ productivity at a wide range of OLRs (1.52–7.5 g chemical oxygen demand [COD]/L/d). Bacterial community analyses were conducted for the three vertical layers using 454 pyrosequencing. The compositions of the bacterial community according to the vertical layers were compared with each other and speculation on the bacterial spatial distribution was performed based on potential roles and characteristics of the bacteria.

## Materials and Methods

### Feedstock and seeding source

Food waste leachate, collected from a local food waste recycling facility located in Daejeon metropolitan city in Korea, was shredded to a diameter of <5 mm using a hammer crusher (TOP‐03H, Hankook Engineering, Yongin, Korea). The shredded leachate was then kept at 4°C in a refrigerator to avoid unintended microbial reactions. As a seeding source, anaerobic sludge was taken from a full‐scale mesophilic anaerobic digester treating sewage sludge located in D city. The characteristics of the seed sludge and the food waste leachate were summarized in Table S1. Food waste leachate was diluted with tap water to maintain target OLR for the bioreactor experiment.

### Reactor configuration and operation

**Figure 1:**
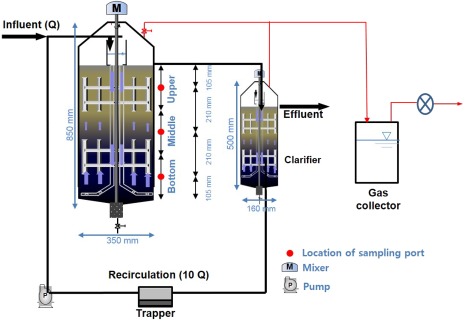
Schematic diagram of the lab‐scale UMAR system.

The mesophilic UMAR consisted of an anaerobic reactor (350 mm diameter and 850 mm height) and a clarifier (160 mm diameter and 500 mm height) with effective volumes of 60 and 8.4 L, respectively (Figure 1). To achieve the desirable OLRs, substrate (*Q*) was semi‐continuously (10 min for every hour, 240 min/d) fed into the anaerobic reactor. Thickened effluent was continuously recirculated (10 *Q*) through a rotating distributor located at the bottom of the UMAR to enhance internal mass transfer. To prevent short‐circuits of the influent and to prevent channeling effects between the substrate and the microorganisms, the distributor and vertical/horizontal baffles were attached to the shaft of the cyclo‐reducer, which rotated at a tip speed of five revolutions per minute.^16^ Both reactor units were inoculated with the seeding sludge and purged with N_2_ gas for 10 min to remove oxygen. After the start‐up period (up to 2.1 g COD/L/d OLR), hydraulic retention time was fixed at 14 days, which corresponds to 0.02 m/h of upflow velocity. The OLR gradually increased to 7.5 g COD/L/d when the practical indices of the steady‐state were stabilized at each OLR: pH, COD removal efficiency, and CH_4_ production rate. To prevent accumulation of mineralized residues inside the system, excessive sludge was periodically removed from the bottom of the clarifier.

### Sampling, DNA extraction and PCR

To analyze the bacterial community structure in the UMAR, sludge samples were taken from three vertical parts of the anaerobic reactor (upper, middle, and bottom) at the end of the experiment (7.5 g COD/L/d OLR). Composite samples were taken at three different horizontal positions and were mixed for each depth. The DNA was extracted using Ultraclean Soil DNA Kit (Mo Bio Laboratories, Solana Beach, CA) and purified using Ultraclean Microbial DNA Isolation Kit (Mo Bio Laboratories) as manufacturers' instructions. A 20‐ng aliquot of each sample DNA was used for a 50‐µl PCR reaction. The 16S universal primers 27F (5′ GAGTTTGATCMTGGCTCAG 3′) and 800R (5′ TACCAGGGTATCTAATCC 3′) were used to amplify the 16s rRNA gene of bacteria.^17^, ^18^ A Fast Start High Fidelity PCR System (Roche Diagnostics, Manheim, Germany) was used for PCR under the following conditions: 94°C for 3 min followed by 35 cycles of 94°C for 15 s, 55°C for 45 s and 72°C for 1 min, and a final elongation step at 72°C for 8 min. The PCR products were purified using AMPure beads (Beckman Coulter, Miami, FL).

### Pyrosequencing and data analysis

The purified PCR products were used to prepare a library according to GS FLX Titanium library preparation guide (Roche) and the library was quantified using the Picogreen assay (Life Technologies, Carlsbad, CA). Pyrosequencing was performed using a GS FLX Titanium (Roche) by a commercial sequencing facility (Macrogen, Seoul, Korea). Sequences were filtered to minimize the effects of poor‐quality sequences using software MOTHUR.^19^ Sequencing errors were minimized by removing sequences with more than one ambiguous base‐call, and by retaining only sequences that were 300 bp or longer.^20^ Sequences were barcode‐sorted and the barcode and primer sequences were trimmed. Operational taxonomic units (OTUs) were defined by a 3% distance level and possible chimeras were removed using the UPARSE pipeline.^21^ The OTUs were phylogenetically classified using Classifier at the Ribosomal Database Project^22^ and confidence value threshold of 50% was used to identify taxa, except for one taxa (*Nitratiruptor*). Heatmap and diversity indices were depicted using software package R employing vegan and ggplot2 libraries. Phylogenic tree was constructed with maximum likelihood method using MEGA 6 software,^23^ and annotated with a heatmap for log transformed relative abundance of OTUs using Evolview v2.^24^ Further, functional profiles of the bacterial communities were predicted using Tax4Fun package.^25^ It works by blasting the OTUs against the SILVA database (SILVA SSU Ref NR database release 115 and KEGG database release 64.0) and then utilizing ultrafast protein classification (UProC) tool^26^ to find metabolic functional profiles for OTUs to generate a [P (OTUs) × K (KEGG K enzymes)] table. Multiplying [N (samples) × P] OTU table with [P × K] OTUs metabolic profile gives a sample‐wise [N × K] table which is not only normalized for 16S rRNA gene copy numbers but also gives relative abundance of KEGG K enzymes within the samples. The sequences reported in this study were deposited in the NCBI Genbank database (accession numbers: KT319842–KT319920).

### Analytical methods

The concentrations of the COD, total solids (TS), volatile solids (VS), total nitrogen, total phosphorus (TP), and alkalinity were measured according to standard methods.^27^ The measured biogas production was adjusted to a standard temperature (0°C) and pressure (760 mmHg). The CH_4_ gas content was analyzed using gas chromatography (GC, SRI 310) equipped with a thermal conductivity detector and a 0.9 m × 3.2 mm stainless steel column packed with a Porapak Q mesh 80/100 with helium as the carrier gas. The temperatures of the injector, detector, and column were maintained at 80, 90, and 50°C, respectively.

## Results

### Performance of UMAR

**Figure 2:**
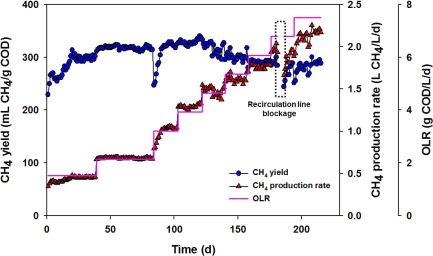
CH_4_ yield and CH_4_ production rate of the UMAR system at different OLRs.

The AD performance of the lab‐scale UMAR at various OLRs is depicted in Figure 2 and Table 1. In general, a stable and effective AD performance was achieved during the long‐term operation (>200 days). The average COD removal efficiency, CH_4_ production rate, and CH_4_ yield at the peak OLR (7.5 g COD/L/d) were 89%, 2.10 L CH_4_/L/d, and 280 mL CH_4_/g COD, respectively. The COD removal efficiencies of 89 − 95% in this study could be compared with to previous studies treating FWL as AD; higher than 73–86% in conventional mesophilic two‐stage continuous stirred tank reactor^7^ and comparable to 93% in combined mesophilic anaerobic‐thermophilic aerobic process.^28^ At 6.79 g COD/L/d OLR, a temporary operational failure was encountered due to blockage of the recirculation line caused by excessively accumulated mineralized residues. Without recirculation, only 50–60% of the usual AD performance (i.e., COD removal efficiency, CH_4_ production rate, and CH_4_ yield) was achieved due to insufficient mixing for the upward plug‐flow stream, highlighting the importance of designed recirculation in the UMAR system. To solve this problem, an additional gadget called a “trapper” was installed in the recirculation line to further remove mineralized residues, which were separated at the bottom of the trapper by gravity; with this device in place, the AD performance soon recovered (Day 185 and on; Figure 2). In short, the structure and function of the UMAR enabled this process to treat solid organic waste (up to 8.5% TS) at high efficiency, which was demonstrated over 200 days of continuous operation. However, excessive accumulation of mineralized residues in the reactors must be avoided to maintain its performance.

**Table 1:** Performance of the UMAR at Different OLRs

Category	Item	Unit	OLR (g COD/L/d)								
			1.5	2.1	3.2	3.9	4.6	5.4	6.1	6.8	7.5
Influent	COD	g/L	23.2	24.5	45.1	55.2	65.0	60.4	68.7	94.6	110.4
	SCOD	g/L	4.1	6.6	17.0	19.5	19.0	20.4	19.8	25.7	34.5
Effluent	pH	‐	8.2	8.1	8.2	8.1	8.1	8.0	8.1	8.0	8.0
	Alkalinity	mg CaCO_3_/L	3,313	3,320	4,323	4,270	4,310	4,620	4,760	4,800	4,850
	${\text{NH}}_{4}^{+}$‐N	mg/L	620	720	1,050	1,210	1,500	1,420	1,850	2,450	2,540
	Total N	mg/L	865	870	1,280	1,520	1,840	2,250	2,750	3,200	3,320
	Total P	mg/L	47	45	60	128	160	185	210	225	245
Performance	COD removal	%	94	95	90	91	90	92	92	90	89
	MY^*^	mL CH_4_/g COD	298	317	321	327	319	302	290	280	280
	MPR^†^	L CH_4_/L/d	0.45	0.68	1.03	1.28	1.48	1.62	1.76	1.90	2.10

*MY, methane yield. †MPR, methane production rate.

### Bacterial community analysis by 454 pyrosequencing

To investigate the spatial distribution of bacteria in the upward plug‐flow reactor, the anaerobic consortia within the UMAR was sampled individually from three vertical sampling ports (upper, middle, bottom). The three ports represent the total sludge contained in the reactor by approximately the equal volumes (Figure 1). The three sludge samples showed negligible difference (<5%) for their physico‐chemical properties, such as pH (8.0–8.1), TS (42.7–43.2 g TS/L) and VS (21.1–21.8 g VS/L) concentration. However, the 454 pyrosequencing results revealed that the bacterial communities were remarkably different along the vertical position.

A total of 16,223 high‐quality sequence reads were obtained from pyrosequencing (Table 2). These sequences were assigned to a total of 72 OTUs belonging to the upper (40 OTUs), the middle (34 OTUs), and the bottom (30 OTUs) samples. The Shannon diversity index was the lowest for the middle sample, while the upper and the bottom samples showed comparable values. Similarly, Pielou's evenness index was the lowest for the middle. The Bray–Curtis similarity was also calculated to show the beta‐diversity among the different parts of the reactor (Table 2). The similarity indices for the upper part were 0.0035 (0.0035 if rarefied) against the middle and 0.0015 (0.0022) against the bottom, whereas the index was 0.2848 (0.3893) between the middle and the bottom. These results indicate that the bacterial communities between the middle and the bottom parts were more similar to each other than they were to that of the upper part, although the middle sample showed a relatively more skewed bacterial community structure out of the three.

**Table 2:** Summary of the Sequencing Results and the Diversity Indices

Sample	Number of High‐Quality Reads	Number of OTUs	Shannon Index	Pielou's Evenness Index	Beta‐Diversity*	
					Middle	Bottom
Upper	6321	40 (39.5)†	2.009 (2.006)	0.545 (0.546)	0.0035 (0.0035)	0.0015 (0.0022)
Middle	6824	34 (32.0)	1.583 (1.583)	0.449 (0.456)	‐	0.2848 (0.3893)
Bottom	3078	30	1.984	0.583	‐	‐

*Beta‐diversity as Bray‐Curtis similarity index. ^†^Numbers in parentheses are the average of 100 trials calculated after rarefying to the least number of reads (i.e., 3078 for the bottom).

**Figure 3:**
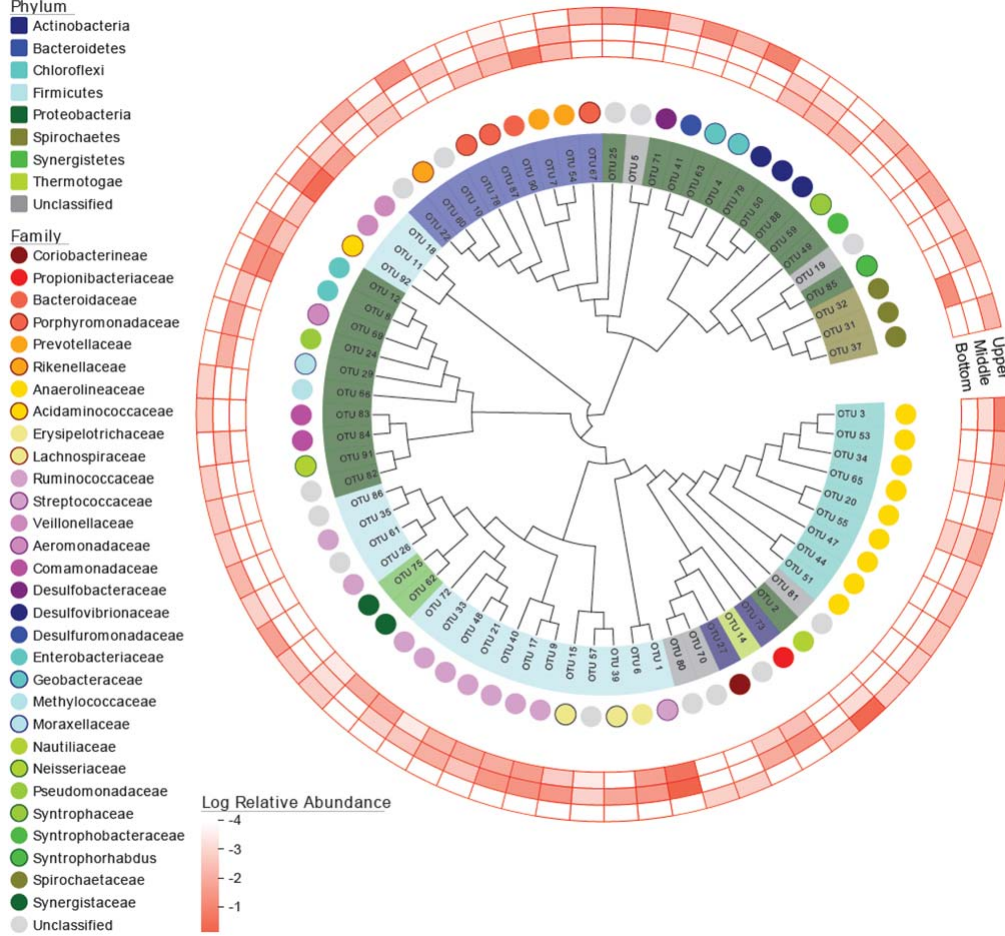
Maximum likelihood tree for 16S rRNA gene sequences of the observed OTUs. Color legends indicate phylogenetic assignment at phylum (shades) and family (circles) levels. Outer rings show log normalized relative abundances for the OTUs in the three samples.

This trend was further visualized with a phylogenetic tree for the OTUs containing annotations for taxonomic affiliations and relative abundance profiles (Figure 3). Among the 72 OTUs, only three (OTUs 20, 62, and 90) were detected in all three samples and five (OTUs 2, 3, 22, 41, and 61) were found in both the middle and the upper. The other 64 OTUs were detected either in a single sample (44 cases) or in both the bottom and the middle (20 cases), indicating no OTU was shared by the bottom and the upper only. These results suggest that the bacterial community structure might have shifted along the axis of the reactor flow (i.e., the vertical axis). A previous study showed that the bottom part (nearest to the substrate inlet) showed more diverged microbial community structure to the inoculum than the upper part in an expanded granular sludge bed, implying the reactor flow may have a significant impact on microbial community development.^15^

**Figure 4:**
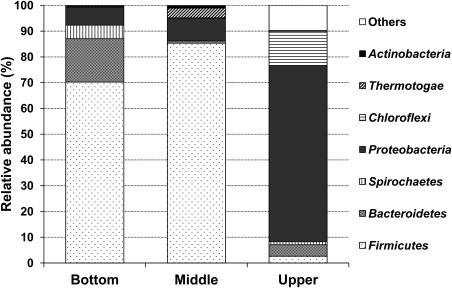
Relative abundance of bacterial phyla at the upper, middle, and bottom part of the UMAR.

**Figure 5:**
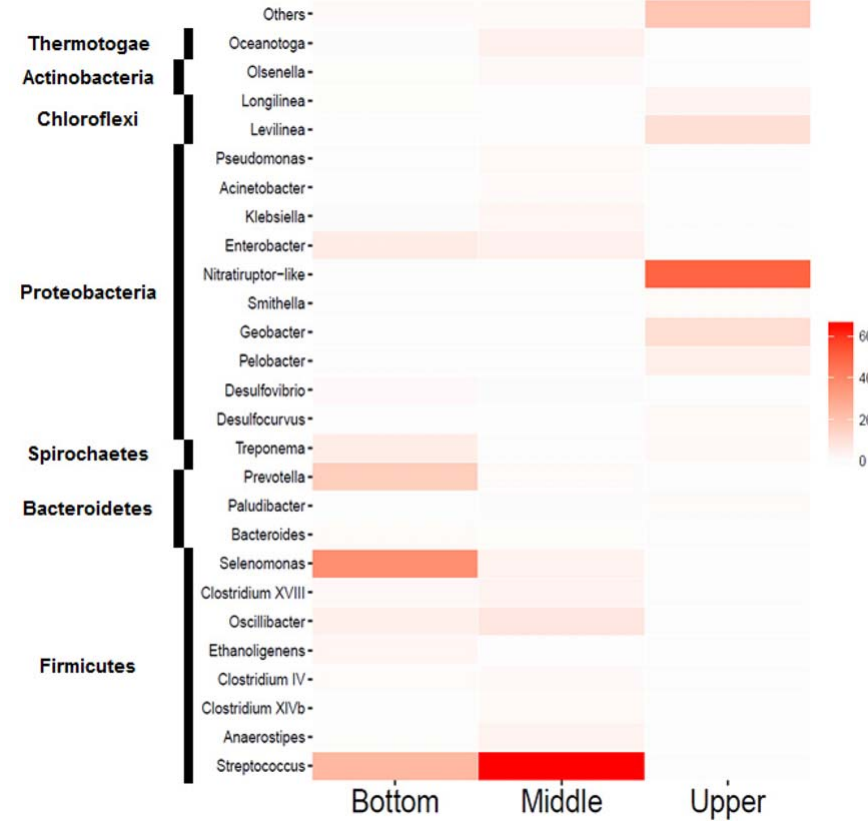
Heatmap displaying the relative abundance of bacterial genera in the samples. Members with average abundance > 0.1% are shown.

The bacterial members in the UMAR were affiliated within 8 identified phyla and 30 identified families (Figures 3 and 4). The majority of the bacterial community at the upper part of the reactor (86.2% relative abundance) was classified into one of the three major phyla: *Proteobacteria* (68.4%), *Chloroflexi* (13.3%), and *Bacteroidetes* (4.5%). In contrast, *Firmicutes* (85.3%), *Proteobacteria* (8.8%), and *Thermotogae* (4.0%) were the most abundant phyla at the middle, while *Firmicutes* (70.2%), *Bacteroidetes* (16.8%), *Proteobacteria* (7.0%), and *Spirochaetes* (5.3%) were at the bottom. A total number of 26 genera were identified at the genus level above the threshold of 0.1% average relative abundance (Figure 5). *Nitratiruptor* (49.7%), *Geobacter* (10.5%), *Levilinea* (10.0%), *Pelobacter* (4.6%), and *Longilinea* (2.7%) were the most abundant groups from the upper sample. For the middle part, *Streptococcus* (65.6%) was the most dominant genus, followed by *Oscillibacter* (7.3%), *Enterobacter* (3.9%), *Oceanotoga* (3.7%), *Selenomonas* (3.4%), *Anaerostipes* (3.3%), and *Clostridium XVIII* (3.1%). Genera *Selenomonas* (37.4%), *Streptococcus* (23.8%), *Prevotella* (15.9%), *Enterobacter* (5.6%), *Treponema* (5.3%), and *Oscillibacter* (4.1%) constituted the majority of the bottom region.

### Prediction of the functional profiles of the bacterial community using Tax4Fun

The Tax4Fun package was used to obtain a further insight into the potential functions associated with the bacterial communities along vertical positions.^25^ Briefly, this method provides additional mapping information of the OTUs derived from the 16S rRNA amplicon sequencing to the functional profiles of the nearest KEGG organisms using a threshold bitscore >1,500 in the BLASTN analysis. This method could be useful to predict the potential functions of the microbial community when only the 16S rRNA‐based sequence data is available but not the metagenomic data. The predicted functional profiles generated by Tax4Fun outperformed those by PICRUSt^29^ when compared with the metagenomic profiles.^25^ Tiers 1–3 KEGG orthology categories with >0.5% average relative abundance are shown in Supporting Information Table S2.^30^ The major functional category identified in the prediction was metabolism, including carbohydrate, amino acid, lipid, nucleotide, cofactors/vitamins, and energy metabolisms. In general, the predicted abundance of the functional groups was similar between the samples. Nonetheless, the apparent abundance of some functional groups was dissimilar between the upper and the other two: carbohydrate metabolism (upper‐to‐others ratio of 89%), lipid metabolism (68%), energy metabolism (154%), replication and repair (81%), and cell motility (207%).

## Discussion

A distinctive feature of the UMAR is its upward plug‐flow stream. This flow regime is hypothesized to allow different layers in a single reactor, presumably leading it to act as a multi‐functional reactor and to harbor various microorganisms with different characteristics. This concept was previously demonstrated in an aerobic equivalent of UMAR, the upflow multi‐layer bioreactor, for an aerobic nutrient removal process (Korean Patent No. 1012405410000).^31^ The UMAR system tested in this study showed a stable performance at an OLR of up to 7.5 g COD/L/d (Table 1). One of the remarkable advantages of UMAR to a typical UASBr was its allowance for applying highly particulate substrate, up to 75.9 g particulate COD/L at the highest OLR. In a typical UASBr, the suspended solids level of the influent is < 1 g/L.^32^ The upward velocity of UMAR (0.02 m/h) is much slower than the typical upflow velocity of about 1.0 m/h for a UASBr,^3^, ^32^ but is similar to that of an anaerobic plug‐flow reactor (0.01 m/h).^33^ The low upflow velocity can reduce the hydraulic shearing force and minimize the detachment of the captured particulate substrates, allowing sufficient contact time for solids organics.^34^ In this regard, employing the plug‐flow‐type upward stream could be beneficial to treat high‐solids wastes such as food waste leachate.

The clarifier in the UMAR system has contributed to the stable reactor performance by ensuring high degree of sludge retention. Because of the highly variable nature of the feedstock, reactor stability is one of the most important concerns for successful operation of an AD process treating high‐solids wastes. The heterogeneity and fluctuation of organic solid feedstock can negatively affect the AD performance because major components of organic waste (i.e., carbohydrate, protein, and lipid) undergo different biochemical pathways yielding potentially unbalanced intermediates at different rates.^35^ For instance, an overloading of easily biodegradable organics such as carbohydrate may cause pH drop due to the imbalance between production and consumption of volatile fatty acids. For the stability recovery, huge efforts are commonly required such as adding buffer and diluting AD reactor with external sludge sources. However, initial stage of reactor instability due to overloading could be adequately managed in this study by enhancing the recirculation rate of the clarifier to the main AD reactor in UMAR.

The pyrosequencing revealed that the UMAR contained diverse bacterial taxa (Figures 3, 4, 5). The bacterial community structures were comparable to previous studies that *Firmicutes*, *Proteobacteria*, and *Bacteroidetes* were the core populations in the AD system.^36^, ^37^ At different levels of the bioreactor, however, the UMAR showed significantly different bacterial community structures (Figure 3). Because the UMAR started with one common seeding source, the developed bacterial community differentiation along the vertical positions can be attributable to the multi‐layered reactor configuration, which has been caused presumably by the plug‐flow stream. In an anaerobic plug‐flow bioreactor, localization of hydrolyzers/fermenters against syntrophs/methanogens could happen according to the hydraulic stream.^33^ The former is likely to be formed near the substrate input point (i.e., substrate distributor), while the latter would be more populated near the end point.

Specifically for this study, the bottom and the middle parts were more alike each other with the Bray‐Curtis similarity index of 0.2848 (0.3898 if rarefied), while the upper was the most distinct sample out of the three (Table 2, Figure 3). The similarity between the bottom and the middle parts can be highlighted by the common dominance by *Firmicutes* in these samples (Figure 4). The predominance of *Firmicutes* in the bottom and the middle can be explained by their versatile roles in the AD system, metabolizing a variety of substrates including proteins, lipids, cellulose, sugars, and amino acids by producing cellulases, lipases, proteases, and other extracellular enzymes.^10^ Although the relative abundance of the members of *Firmicutes* were different between the bottom and the middle (Figure 3), they shared most of the *Firmicutes*‐affiliated genera identified (Figure 5). The gene functions predicted by Tax4Fun showed little distinction between the bottom and the middle (Supporting Information Table S2). The higher abundance of genes related to carbohydrate metabolism (average 112% compared with the upper) and lipid metabolism (148%) imply that the bottom‐to‐middle part of the UMAR was populated by bacterial populations, such as *Firmicutes*, which can hydrolyze and ferment crude organics. The heterogeneity of microbial community structures along the vertical axis confirms that the hydraulic regime of the UMAR, including the recirculation at 10 *Q*, led to a multi‐layered system but not a homogeneous mixing.

In contrast, the upper part of the UMAR was dominated by *Proteobacteria* (Figures 3 and 4). This result, linked with the near absence of *Firmicutes* in the upper region, might be the reason why energy metabolism, such as methane and sulfur metabolisms, were more pronounced in the upper sample (Supporting Information Table S2). The genus *Geobacter*, *Desulfovibrio*, *Syntrophobacter*, and *Syntrophorhabdus*, represented within *Deltaproteobacteria*, are commonly associated with syntrophic bacteria.^38^ Syntrophy, which is mutually beneficial to the participants in metabolic processes, thermodynamically plays very important roles in the AD process. The biological oxidation of other fatty acids (propionate, lactate, and butyrate) to acetate is thermodynamically unfavorable under standard conditions; however, it becomes favorable when feeding partners such as methanogens consume the intermediates, keeping them at low concentrations. Besides inducing the oxidations of various fatty acids to acetate, syntrophic bacteria are also capable of metabolizing hydrocarbons, which are known to be relatively inert and even toxic materials.^39^ *Syntrophobacter* spp. is the most common propionate oxidizer that can degrade propionate to acetate and CO_2_ using the methylmalonyl‐CoA pathways; *Smithella* spp., another common oxidizer, uses the condensational method.^40^ *Syntrophorhabdus* spp. has been reported to be capable of utilizing phenol, p‐cresol, isophthalate, benzoate, and 4‐hydroxybenzoate with hydrogenotrophic methanogen via syntrophic reaction.^41^

This could be linked to the relative dominance of *Firmicutes* in the middle and the bottom parts (Figure 5). The predominance of metabolically versatile *Firmicutes* in the middle and the bottom could presumably have led to an increase of H_2_ flux. Although simultaneous H_2_ consumption is anticipated in such a situation, the H_2_ concentration might have been higher in the middle and the bottom layers. Assuming that, the upper part of the reactor should be thermodynamically more preferable for syntrophic bacteria within the *Deltaproteobacteria*. It could be inferred that the syntrophic bacteria and their partner methanogens could have occurred in higher abundance in the upper part, where easy substrates for methanogenesis, such as acetate, are likely depleted due to the longer residence time from the substrate inlet. *Methylomonas*, a methanotrophic bacteria, was also found exclusively in the upper sample. Methanotrophs are unique in their ability to oxidize and utilize CH_4_ as a sole carbon and energy source; *Methylomonas* is known as a Type I variety of the methanotrophs, which use the ribulose monophosphate pathway to assimilate carbon.^42^ The limited appearance of *Methylomonas* in the upper part could presumably be attributable to CH_4_ availability due to the extremely low solubility of CH_4_ in water that leads to an equilibrium towards the gas phase at the gas‐liquid phase. In addition, it is difficult to speculate on the effects of *Methylomonas* on AD performance because both a negative effect from CH_4_ consumption and a positive effect from the degradation of toxic chlorinated hydrocarbon can be expected.^43^

The relatively diverse appearance of *Bacteroidetes* in the bioreactor can be linked to the protein metabolism; these bacteria are well known as proteolytic bacteria that participate in the degradation of proteins, and are capable of fermenting amino acids to acetate and ammonia.^44^ This speculation was in accordance with the very similar amino acid metabolism levels between the three samples, as predicted by the Tax4Fun pipeline (Supporting Information Table S2). The phylum *Chloroflexi* was mainly found in the upper part of the reactor (Figures 3 and 4). Despite the frequent observation of *Chloroflexi* in various AD systems, their functions are still unclear.^36^, ^37^, ^45^ Yamada et al.^46^ suggested that the functions of *Chloroflexi* in the AD process are carbohydrate degradation and cellular matter degradation; in addition, their glucose‐degrading functions were also demonstrated.^45^

Although this study successfully investigated the bacterial community structures in a novel AD platform, the UMAR, the lack of archaeal counterpart data remains as a potential limitation to the full understanding of the system. Among anaerobic archaea, methanogens perform a unique function of producing CH_4_ in AD and are recognized as the major archaeal group in most AD bioreactors.^14^, ^47^ In previous studies, anaerobic digesters treating food waste leachate were often populated by mixotrophic *Methanosarcinales* and hydrogenotrphic *Methanomicrobiales* and *Methanobacteriales*.^7^, ^28^, ^48^ Although no direct data is given for the UMAR, considering the active CH_4_ production throughout the operation (Figure 2), a similar methanogen structure could be anticipated, for example, localization of active hydrogenotrophic methanogens in the upper part along with syntrophic *Deltaproteobacteria* (Figures 3, 4, 5). A further study is required to elucidate the structures and functions of the archaeal‐bacterial communities in this system.

## Conclusions

The UMAR system was developed as a high‐rate anaerobic system to treat solid organic wastes, and its anaerobic performance was successfully demonstrated during a continuous operation. As hypothesized, the effects of the upward plug‐flow stream on the spatial distribution of bacterial communities at different vertical locations (upper, middle, bottom) were clearly demonstrated using the 454 pyrosequencing technique. Generation of the different zones in the UMAR seems to have allowed various bacteria to live in their preferable conditions, presumably resulting in effective AD performance.

## Supporting information

Supporting Information.
