# Supplementary material for: Bacterial community analysis in upflow multilayer anaerobic reactor treating high‐solids organic wastes
Source: Biotechnol Prog. 2017 Sep 6;33(5):1226–34. doi: 10.1002/btpr.2540 (PMC6585729; doi:10.1002/btpr.2540)
Supplement: Supplementary file 1 — Supporting Information. [file BTPR-33-1226-s001.docx]

***Biotechnology Progress***

**Bacterial community analysis in upflow multi-layer anaerobic reactor (UMAR) treating high-solids organic wastes**

Si-Kyung Cho^a^, Kyung-Won Jung^b^, Dong-Hoon Kim^c^, Joong-Chun Kwon^d^, Umer Zeeshan Ijaz^e^, and Seung Gu Shin^f,*^

^a^ Department of Biological and Environmental Science, Dongguk University, 32 Dongguk-ro, Ilsandong-gu, Goyang, Gyeonggi-do, Republic of Korea

^b^ Center for Water Resources Cycles Research, Korea Institute of Science and Technology, P.O. Box 131, Cheongryang, Seoul 130-650, Republic of Korea

^c^ Department of Civil Engineering, Inha University, 100 Inharo, Nam-gu, Incheon, Republic of Korea

^d^ Ecodigm, 10-6, 339 Expo-ro, Yuseong-gu, Daejeon, Republic of Korea

^e^ Infrastructure and Environment Division, School of Engineering, University of Glasgow, Glasgow, G12 8LT, UK

^f^ School of Environmental Science and Engineering, Pohang University of Science and Technology, 77 Cheongam-ro, Pohang, Gyeongbuk, Republic of Korea

Running headline: Bacterial community in UMAR system

^*^Correspondence: +82-54-279-8317, +82-54-279-8299 (fax), candlit@postech.ac.kr

**Table S1**

**Characteristics of the seed sludge and the food waste leachate.**

| Item | Unit | Seed sludge | Food waste leachate |
| --- | --- | --- | --- |
| pH | - | − | 4.5 ± 0.3 |
| Alkalinity | mg CaCO_3_/L | 1850 | − |
| Total COD | g/L | − | 147.7 ± 26.7^*^ |
| TS | g/L | 21 | 113 ± 14 |
| VS | g/L | 14 | 100 ± 15 |
| TN | mg-N/L | − | 3745 ± 797 |
| TP | mg-P/L | − | 1017 ± 394 |
| Ammonia | mg NH_4_^+^-N/L | − | 473 ± 346 |

^*^ Mean ± standard deviation.

**Table S2**

The Tax4Fun prediction of the tiers 1, 2, and 3 KEGG orthology (KO) categories (> 0.5% of the total) derived from the bacterial 16S rRNA amplicon sequencing.

| KO categories | Bottom | Middle | Upper |
| --- | --- | --- | --- |
| **Metabolism** | **63.8** | **63.9** | **64.8** |
| **Carbohydrate metabolism** | **18.4** | **19.6** | **17.0** |
| ko00010 Glycolysis / Gluconeogenesis | 2.3 | 2.6 | 2.2 |
| ko00020 Citrate cycle (TCA cycle) | 1.4 | 1.2 | 1.6 |
| ko00030 Pentose phosphate pathway | 1.1 | 1.3 | 1.3 |
| ko00051 Fructose and mannose metabolism | 1.0 | 1.0 | 0.8 |
| ko00052 Galactose metabolism | 1.2 | 1.4 | 0.7 |
| ko00500 Starch and sucrose metabolism | 1.7 | 2.0 | 1.2 |
| ko00520 Amino sugar and nucleotide sugar metabolism | 2.2 | 2.1 | 2.1 |
| ko00620 Pyruvate metabolism | 2.4 | 2.7 | 2.6 |
| ko00630 Glyoxylate and dicarboxylate metabolism | 1.4 | 1.1 | 1.3 |
| ko00640 Propanoate metabolism | 1.2 | 1.3 | 1.0 |
| ko00650 Butanoate metabolism | 1.4 | 1.5 | 1.2 |
| ko00020 Citrate cycle (TCA cycle) | 1.4 | 1.2 | 1.6 |
| **Energy metabolism** | **7.4** | **6.5** | **10.7** |
| ko00190 Oxidative phosphorylation | 1.4 | 1.1 | 2.4 |
| ko00710 Carbon fixation in photosynthetic organisms | 0.9 | 0.9 | 1.2 |
| ko00720 Carbon fixation pathways in prokaryotes | 2.0 | 1.7 | 2.7 |
| ko00680 Methane metabolism | 1.3 | 1.3 | 2.4 |
| ko00910 Nitrogen metabolism | 1.0 | 0.7 | 0.8 |
| ko00920 Sulfur metabolism | 0.4 | 0.4 | 0.8 |
| **Lipid metabolism** | **2.7** | **3.3** | **2.0** |
| ko00061 Fatty acid biosynthesis | 0.7 | 0.7 | 0.7 |
| ko00564 Glycerophospholipid metabolism | 0.6 | 0.7 | 0.5 |
| **Nucleotide metabolism** | **8.1** | **8.1** | **7.4** |
| ko00230 Purine metabolism | 4.4 | 4.4 | 4.2 |
| ko00240 Pyrimidine metabolism | 3.7 | 3.6 | 3.2 |
| **Amino acid metabolism** | **11.5** | **11.3** | **11.3** |
| ko00250 Alanine, aspartate and glutamate metabolism | 1.9 | 1.7 | 1.7 |
| ko00260 Glycine, serine and threonine metabolism | 1.5 | 1.5 | 1.4 |
| ko00270 Cysteine and methionine metabolism | 1.7 | 1.6 | 1.4 |
| ko00280 Valine, leucine and isoleucine degradation | 0.6 | 0.6 | 0.4 |
| ko00290 Valine, leucine and isoleucine biosynthesis | 0.7 | 0.7 | 0.9 |
| ko00300 Lysine biosynthesis | 0.9 | 0.9 | 0.9 |
| ko00220 Arginine biosynthesis | 0.8 | 0.8 | 0.9 |
| ko00330 Arginine and proline metabolism | 0.6 | 0.6 | 0.6 |
| ko00340 Histidine metabolism | 0.5 | 0.5 | 0.6 |
| ko00400 Phenylalanine, tyrosine and tryptophan biosynthesis | 1.0 | 0.9 | 1.3 |
| **Metabolism of other** | **2.4** | **2.5** | **1.9** |
| ko00450 Selenocompound metabolism | 0.7 | 0.7 | 0.7 |
| **Glycan biosynthesis and metabolism** | **2.8** | **2.5** | **2.5** |
| ko00550 Peptidoglycan biosynthesis | 1.4 | 1.5 | 1.5 |
| **Metabolism of cofactors and vitamins** | **6.0** | **4.9** | **6.6** |
| ko00730 Thiamine metabolism | 0.7 | 0.6 | 0.7 |
| ko00760 Nicotinate and nicotinamide metabolism | 0.6 | 0.6 | 0.5 |
| ko00770 Pantothenate and CoA biosynthesis | 0.8 | 0.7 | 0.9 |
| ko00780 Biotin metabolism | 0.5 | 0.4 | 0.6 |
| ko00790 Folate biosynthesis | 0.5 | 0.4 | 0.7 |
| ko00670 One carbon pool by folate | 0.8 | 0.7 | 0.7 |
| ko00860 Porphyrin and chlorophyll metabolism | 1.2 | 0.6 | 1.6 |
| **Metabolism of terpenoids and polyketides** | **1.5** | **1.7** | **1.6** |
| ko00900 Terpenoid backbone biosynthesis | 0.7 | 0.7 | 0.6 |
| **Biosynthesis of other secondary metabolites** | **1.7** | **1.5** | **2.0** |
| **Xenobiotics biodegradation and metabolism** | **1.4** | **2.0** | **1.8** |
| **Genetic Information Processing** | **14.5** | **14.1** | **13.1** |
| **Transcription** | **0.6** | **0.6** | **0.6** |
| ko03020 RNA polymerase | 0.6 | 0.6 | 0.6 |
| **Translation** | **4.3** | **4.1** | **4.4** |
| ko03010 Ribosome | 1.4 | 1.3 | 1.5 |
| ko00970 Aminoacyl-tRNA biosynthesis | 2.8 | 2.6 | 2.7 |
| **Folding, sorting and degradation** | **2.6** | **2.4** | **2.4** |
| ko03060 Protein export | 0.8 | 0.8 | 0.8 |
| ko03018 RNA degradation | 1.3 | 1.2 | 1.0 |
| **Replication and repair** | **7.1** | **7.0** | **5.7** |
| ko03030 DNA replication | 1.4 | 1.4 | 1.1 |
| ko03410 Base excision repair | 0.7 | 0.7 | 0.5 |
| ko03420 Nucleotide excision repair | 1.3 | 1.2 | 1.2 |
| ko03430 Mismatch repair | 1.8 | 1.8 | 1.4 |
| ko03440 Homologous recombination | 1.8 | 1.9 | 1.4 |
| **Environmental Information Processing** | **8.3** | **9.5** | **8.1** |
| **Membrane transport** | **4.8** | **5.8** | **3.4** |
| ko02010 ABC transporters | 3.3 | 3.9 | 2.4 |
| ko02060 Phosphotransferase system (PTS) | 0.6 | 1.1 | 0.0 |
| ko03070 Bacterial secretion system | 0.9 | 0.8 | 0.9 |
| **Signal transduction** | **3.5** | **3.8** | **4.7** |
| ko02020 Two-component system | 2.9 | 3.0 | 4.0 |
| **Cellular Processes** | **5.8** | **5.1** | **7.7** |
| **Cell motility** | **1.7** | **1.0** | **2.8** |
| ko02030 Bacterial chemotaxis | 1.1 | 0.6 | 1.6 |
| ko02040 Flagellar assembly | 0.7 | 0.4 | 1.3 |
| **Cell growth and death** | **1.0** | **0.8** | **1.4** |
| ko04112 Cell cycle - Caulobacter | 0.9 | 0.8 | 1.1 |
| **Cellular community - prokaryotes** | **2.8** | **3.0** | **3.3** |
| ko02024 Quorum sensing | 2.2 | 2.5 | 2.1 |
| **Organismal Systems** | **2.8** | **2.4** | **2.4** |
| **Endocrine system** | **1.2** | **1.1** | **0.8** |
| **Aging** | **0.6** | **0.6** | **0.8** |
| **Human Diseases** | **4.7** | **4.9** | **4.0** |
| **Cancers** | **0.9** | **0.9** | **0.4** |
| **Endocrine and metabolic diseases** | **0.5** | **0.5** | **0.5** |
| **Infectious diseases** | **1.4** | **1.4** | **1.1** |
| **Drug resistance** | **1.8** | **1.9** | **1.6** |
| ko01501 beta-Lactam resistance | 0.7 | 0.9 | 0.7 |
